# Differential contribution of transcriptomic regulatory layers in the definition of neuronal identity

**DOI:** 10.1038/s41467-020-20483-8

**Published:** 2021-01-12

**Authors:** Kevin C. H. Ha, Timothy Sterne-Weiler, Quaid Morris, Robert J. Weatheritt, Benjamin J. Blencowe

**Affiliations:** 1 Donnelly Centre for Cellular and Biomolecular Research, University of Toronto, Toronto, ON M5S 3E1 Canada; 2 Department of Molecular Genetics, University of Toronto, Toronto, ON M5A 1A8 Canada; 3 Vector Institute, Toronto, ON M5G 1M1 Canada; 4 Department of Computer Science, University of Toronto, Toronto, ON M5S 4G4 Canada; 5 EMBL Australia, Garvan Institute of Medical Research, Darlinghurst, NSW 2010 Australia; 6 St. Vincent Clinical School, University of New South Wales, Darlinghurst, NSW 2010 Australia; 7 Present Address: BioSymetrics, Inc., Huntington, New York, NY USA

**Keywords:** Gene regulatory networks, RNA splicing, RNA splicing, Neuroscience

## Abstract

Previous transcriptomic profiling studies have typically focused on separately analyzing mRNA expression, alternative splicing and alternative polyadenylation differences between cell and tissue types. However, the relative contribution of these three transcriptomic regulatory layers to cell type specification is poorly understood. This question is particularly relevant to neurons, given their extensive heterogeneity associated with brain location, morphology and function. In the present study, we generated profiles for the three regulatory layers from developmentally and regionally distinct subpopulations of neurons from the mouse hippocampus and broader nervous system. Multi-omics factor analyses revealed differing contributions of each transcriptomic layer in the discrimination of neurons based on their stage of development, region, and function. Importantly, profiles of differential alternative splicing and polyadenylation better discriminated specific neuronal subtype populations than gene expression patterns. These results provide evidence for differential relative contributions of coordinated gene regulatory layers in the specification of neuronal subtypes.

## Abstract (web summary)

Post-transcriptional gene regulation is an important contributor to cell type-specific differences at the transcriptomic level. Here, the authors use a multiomics approach to characterize neuronal diversity in the mouse nervous system, analyzing the relative contributions of multiple layers of transcriptomic regulation in the specification of cell type identity.

## Introduction

The human and mouse brain are composed of ~86 billion^1,2^ and 71 million^3^ neurons, respectively. Furthermore, it has been estimated that neurons can contain thousands to tens of thousands of synapses that are capable of forming billions to trillions of synaptic connections^4,5^. In contrast, *C. elegans* possesses 302 neurons that form ~7500 synaptic connections^6^. These findings and the observation that metazoan species have comparable numbers of protein coding genes highlight the importance of the evolution of gene regulatory complexity as a determinant of neuronal diversity. Neurons are categorized into different subtypes depending on a multitude of related factors, including molecular composition, morphology, location, and physiology^7^, and hundreds of distinct subtypes have been defined in the mammalian brain^8^. Previous efforts directed at classifying neuronal subtypes using high-throughput RNA sequencing (RNA-seq), most recently at a single-cell level, have largely focused on profiling gene expression (GE) changes^9^.

Alternative splicing (AS) and alternative polyadenylation (APA) play numerous critical and multifaceted roles in the vertebrate nervous system^10–12^. These forms of post-transcriptional gene regulation generate multiple transcript isoforms from the same gene by differential selection of splice sites and 3′ end poly(A) sites, respectively. Both mechanisms are widespread in multicellular eukaryotes; more than 90% of human genes produce transcripts that are alternatively spliced^13,14^ and ~80% of human genes have multiple poly(A) sites^15^. The importance of coordinated regulation of neural-differential AS is highlighted by the concentration of AS programs in genes that function in key processes associated with nervous system development and function, such as neurogenesis, axonogenesis, synaptic biology, neurotransmitter trafficking, and signaling. It has been estimated that approximately one-third of neural-regulated exons function in the remodeling of protein–protein interaction networks^16^, with many belonging to a class of neural microexons that are less than 27 nt in length^17^. Moreover, to-date, dozens of individual neural-differential exons, and the trans-acting factors that control them, have been functionally characterized and shown to play critical roles in these processes^18^.

Cell type- and tissue-differential GE patterns have been largely conserved during vertebrate evolution, whereas differential AS patterns, overall (with the exception of conserved sub-networks of AS events) have evolved rapidly and display increased complexity during evolution, particularly in the nervous system^19,20^. Moreover, increasing examples have been reported of functionally important neuronal subtype-specific AS patterns^21–23^. APA also shows extensive variation between tissues and species^24,25^, with pronounced changes in neural cells that predominantly result in longer 3′ UTRs to facilitate extensive post-transcriptional regulation^24,25^. For example, changes in the expression of 3′ UTR sequences through APA can regulate the localization of messenger RNAs (mRNAs) to dendrites and axons, in part to facilitate localized translation^26^. APA has been implicated in the regulation of long-term potentiation of hippocampal neurons^27^. Hence, in conjunction with differential GE, AS and APA are important and complementary regulatory layers that contribute to the specification of neuronal subtypes. Yet, the relative degrees to which these regulatory layers contribute to cell-type specification in the nervous system has not been systematically investigated.

In this study, we investigate how steady-state GE, AS, and APA changes are coordinated in defining different classes of neurons using high-throughput RNA sequencing (RNA-seq) data. To this end, we first analyzed RNA-seq data^28^ generated from pyramidal neurons purified from the discrete regions of the mouse hippocampus, including proximal-distal, dorsal-ventral, and superficial-deep axes of CA1 and CA3 subfields. In addition to recapitulating previously observed^28^ GE differences between dorsal and ventral CA1 pyramidal neurons, we show that differential AS and APA account for a significant proportion of the variation between populations of this class of hippocampal neuronal subtype. In particular, differential AS predominantly accounts for transcriptomic variation between the proximal-distal axis, and significantly contributes to patterns distinguishing regionally separated CA1 and CA3 neurons, whereas differential APA predominantly accounts for transcriptomic variation between pyramidal neurons from other regions of the hippocampus. We next expanded our analysis to neurons across the nervous system, which identified co-regulated patterns of GE, AS, and APA that, to differing extents, discriminate neurons based on their age, region, and function. While genes affected by each of the three regulatory layers were concentrated in different subsets of genes with overlapping functional categories, in certain cases AS and APA uniquely controlled programs depending on the specific neuronal population. Taken together, our results highlight how GE, AS, and APA patterns collectively contribute to the shaping of the transcriptomes that drive neuronal specification. These findings highlight the importance of integrating multiple types of measurements from the same transcriptomic data to establish the molecular underpinnings of cell-type identity.

## Results

### Multiple transcriptomic regulatory layers contribute to neuronal diversity in the hippocampus

**Fig. 1:**
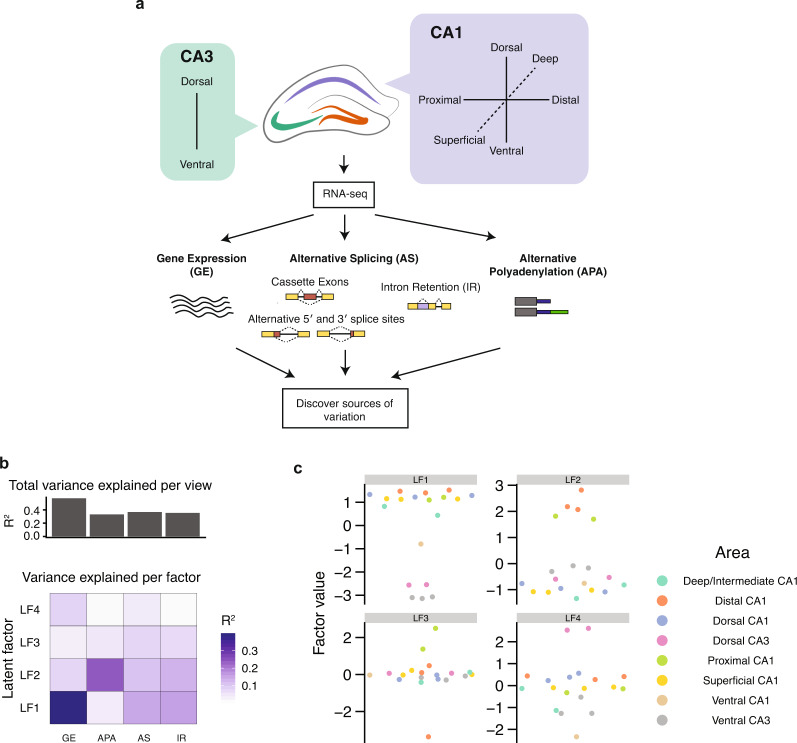
Multi-layered RNA-seq analysis for investigating neuronal subtype diversity. **a** Schematic of the mouse hippocampus (center) and the various anatomical regions assayed by RNA-seq from Cembrowski et al.^28^. The hippocampus is broadly segmented into three subfields: CA1 (purple), CA3 (green), and DG (orange, unlabeled). Computational methods were applied to generate multi-dimensional profiles of each transcriptome, including gene expression (GE), exon sequence alternative splicing (AS), intron retention (IR), and alternative polyadenylation (APA). Hippocampus schematic by Rayna Harris^77^, distributed under a Creative Commons Attribution License. **b** MOFA was applied to investigate the sources of variation that describe the differences between neuronal types. The total contributions of each layer, measured by total variance explained (*R*^2^), are summarized in the bar-plot (top panel). The individual contributions of each layer to each latent factor (LF) are summarized in the heatmap (bottom panel). **c** Beeswarm plots (one-dimensional scatterplots) illustrating the variation described by the top four latent factors inferred by MOFA. Each sample is assigned a factor weight (*y*-axis) signifying its involvement in explaining the factor. MOFA captures sources of variability found between different regions (color).

The hippocampus is a critical and extensively studied brain region with important roles in the limbic system, such as regulating memory formation, behavior, and emotion. Hippocampal neurons are spatially organized into regions or subfields, i.e., the *Cornu Ammonis* fields CA1, CA2, CA3, and dentate gyrus (DG). Increasing evidence supports functional differences between and within subfields^29,30^, yet the molecular determinants underlying these differences are not well understood. To investigate this question at the transcriptomic level, we analyzed RNA-seq data^28^ from excitatory pyramidal neurons isolated from different CA1 and CA3 regions of the mouse hippocampus, including the dorsal, proximal-distal, and ventral subsections (Fig. 1a and Supplementary Data 1). We applied Salmon^31^ to profile GE, VAST-TOOLS^17,32^ and Whippet^33^ to profile AS, and QAPA^34^ to profile APA (Fig. 1a). In this analysis, AS differences were separately quantified for exon sequence-containing events (i.e., simple and complex cassette exons, microexons, and alternative 5′ and 3′ splice sites) and intron retention (IR) events.

To initially investigate global patterns in the data, principal component analysis (PCA) was applied to each individual RNA-Seq analysis output. This analysis distinguishes datasets based on regional identities of neuronal subpopulations (Supplementary Fig. 1a). For example, in addition to clear GE differences between CA1 and CA3 regions, there were clusters corresponding to dorsal and ventral CA1 regions, in line with previous observations^28^. Furthermore, APA, AS, and IR differences captured additional variation between subtypes. For example, APA differences defined a distinct cluster of neurons belonging to the proximal-distal axis, which is further explored below. Taken together, these observations provide evidence that different layers of post-transcriptional regulation differentially contribute to the spatial identity of hippocampal pyramidal neurons.

### Relative contributions of differential gene expression, alternative splicing, and alternative polyadenylation in the definition of neuronal subtypes

To further investigate how GE, AS, and APA are coordinated in the definition of temporally and spatially distinct subpopulations of pyramidal neurons, we employed multiomics factor analysis (MOFA)^35^, a statistical framework for identifying principal sources of variation from multiple data types in an unsupervised manner. Briefly, MOFA jointly analyzes multiple data types using group factor analysis to infer a set of hidden, latent factors unobserved by the data^35^. In a manner similar to PCA, each latent factor represents a component of the unobserved structure of the data that is jointly dependent on multiple input data sources. MOFA has distinct advantages over other multiomics approaches, as it can handle missing values, integrate data from multiple modalities, is robust to differences in measurement scales across inputs, and identify hidden sub-groups underlying the data, for example, those that explain biological and technical sources of variablilty^35^. In the present study, we sought to identify distinct neuronal populations based on one or more underlying data modalities. By interrogating the latent factor scores or loadings assigned by MOFA, the variation explained by each latent factor could be interpreted and annotated, as described below.

To establish a MOFA model, the same set of features analyzed by PCA—that is, quantitative profiles of GE, AS, and APA events—were used as training data (see “Methods” for details). Overall, MOFA inferred four latent factors (LF1, LF2, LF3, and LF4), each of which have shared and unique contributions from each transcriptomic regulatory layer (Fig. 1b). In total, GE accounted for the majority of the total variance (*R*^2^ = 0.57), followed by exonic AS (*R*^2^ = 0.37), IR (*R*^2^ = 0.35), and APA (*R*^2^ = 0.33). These factors describe variability in line with the results from PCA (Fig. 1c and Supplementary Fig. 1a), yet afford a quantitative assessment of the contributions of each layer to neuronal identity. Using the output from MOFA we further investigated LF1 and LF2, which describe regional differences between CA1 and CA3 neurons, and between-axes differences, respectively.

### Contributions of differential gene expression and alternative splicing to regional differences between hippocampal CA1 and CA3 subfields

We performed an in-depth examination of LF1, in which GE, and AS contribute to regional differences between CA1 and CA3 pyramidal neurons (Fig. 1c). To quantify the correlation between a feature (e.g., differential GE or AS event) from each layer and the variation described by a factor, MOFA assigns each feature a score or loading. Hence, loadings with larger magnitudes indicate a stronger correlation or anti-correlation with a factor. To confirm that LF1 describes variation between CA1 and CA3, we compared the loadings assigned to features in the GE layer with the log_2_ fold changes in GE (Supplementary Fig. 1b). Indeed, we observe a strong correlation between these two measurements (Pearson correlation *R* = 0.9, *p* < 2.2 × 10^−16^).

**Fig. 2:**
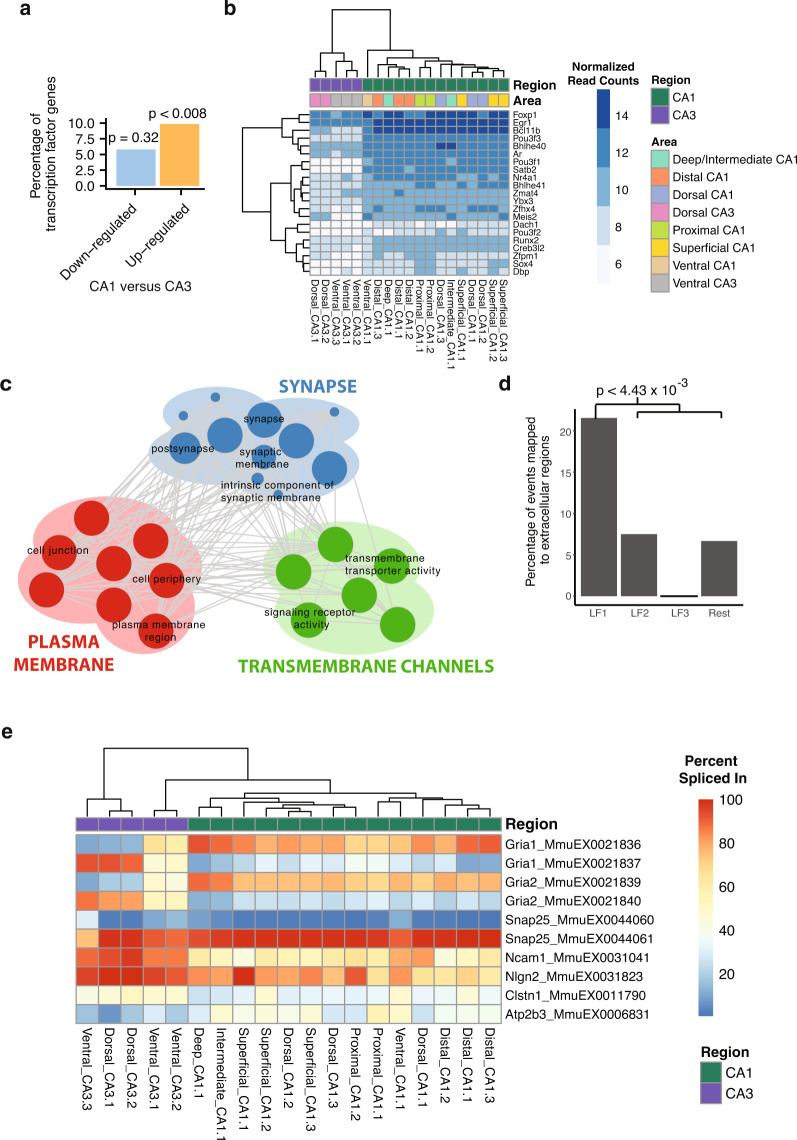
AS in LF1 distinguishes CA1 and CA3 subfields. **a** Bar-plot summarizing the proportion of down- and upregulated genes in CA1 versus CA3 that are annotated transcription factors. Among 298 upregulated genes, the overlap with transcription factors (*n* = 27) was statistically significant (*p* = 0.00844, hypergeometric test). **b** Heatmap showing normalized read counts of the upregulated transcription factors (rows) for each sample (columns). Each sample is annotated according to region and area (horizontal bars above heatmap). The final number in name indicates the replicate number. **c** Enrichment map^74^ for GO, REACTOME and KEGG functional categories of genes from the top AS events with the largest absolute LF1 loading. Node size is proportional to the number of genes associated with the GO category, and edge width is proportional to the number of genes shared between GO categories. **d** Overlap of AS events mapped to extracellular regions for each latent factor (*p* = 4.43 × 10^−3^, two-sided Fisher’s exact test). **e** Heatmap showing the percent spliced-in (PSI) values of top AS events from LF1. These events include pairs of mutually exclusive cassette exons in Gria1 and Gria2 (rows), which are differentially spliced between CA1 and CA3 regions (compare left CA3 cluster with right clusters). Row names consist of gene name and VAST-TOOLS event IDs. Sample naming is as in Fig. 2b.

Next, to investigate whether the top scoring features associated with LF1 are enriched for specific biological functions, we performed Gene Ontology (GO) analysis for each regulatory layer using the set of input features as background. Interestingly, significant GO term enrichment was observed for GE and AS. For GE, we observe enrichment for the GO term “transcription factor activity” (FDR < 0.05, FDR-corrected hypergeometric test). This prompted us to ask whether the top genes are enriched for annotated transcription factors among a recently curated list of 1,816 transcription factors^36^. Indeed, of 213 genes that were upregulated in CA1 neurons as detected by DESeq2^37^ ($\left| {{\mathrm{log}}_2\phi } \right| \, > \, 1$, FDR < 0.05, where $\phi$ is the fold change in gene expression between CA1 and CA3), 21 are annotated transcription factors, which represents a significant enrichment (*p* = 0.00844, hypergeometric test; Fig. 2a, b).

In the AS (exonic sequence events) layer, we identified genes encoding exons with differential splicing between CA1 and CA3, as measured by the change in Percent Spliced In (∆PSI) values. As validation, the loadings assigned to the AS events were consistent with detected ∆PSI differences between CA1 and CA3 (Supplementary Fig. 1c, d). The genes representing the top loaded events are enriched in GO terms associated with synaptic vesicles, plasma membranes, and the cell surface (Fig. 2c, *p* < 0.05, FDR-corrected hypergeometric test). Confirming the association of these AS events in genes encoding membrane proteins, protein sequences encoded by these exons showed strong enrichment for overlap with extracellular regions (Fig. 2d, *p* = 4.43 × 10^−3^, two-sided Fisher’s exact test).

Interestingly, among the AS events with the top weights assigned by MOFA, we identified previously validated mutually exclusive alternative exons in the *α*. -amino-3-hydroxy-5-methyl-4-isoxazolepropionic acid (AMPA) receptor genes *Gria1* and *Gria2* that display differential splicing between CA1 and CA3^38^ (Fig. 2e). In addition, we identified mutually exclusive exons in *Snap25* and an alternative VASE (VAriable domain Spliced Exon) exon in *Ncam1*; notably both of these genes have important roles in synaptic plasticity and the inhibition of neurite outgrowth, respectively^39,40^. These results demonstrate that our analytical approach using MOFA to analyze the RNA-seq data described above identifies new, as well as previously validated, transcriptomic differences between subregions of the hippocampus.

### Differential expression of ribosomal protein genes distinguishes the proximal-distal axes of hippocampus

**Fig. 3:**
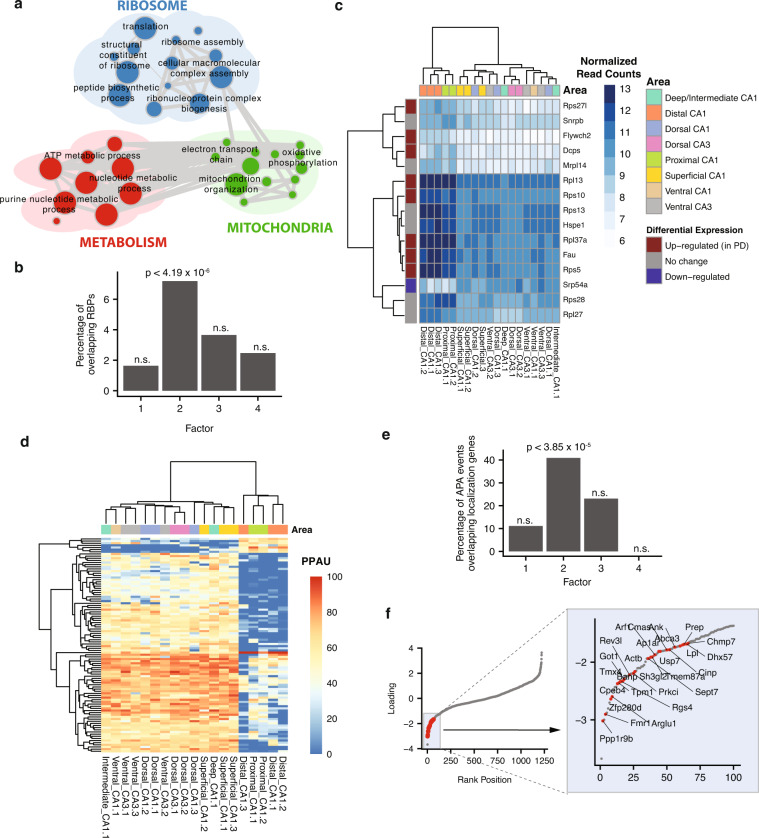
GE and APA changes in LF2 distinguish proximal-distal neurons from other axes. **a** Enrichment map displaying the functional enrichment analysis of genes from top GE events with the largest absolute LF2 loading. See Fig. 2c for description of enrichment maps. **b** Bar-plot showing the proportion of top-weighted genes that overlap known RNA-binding proteins (RBPs). When comparing the degree of overlap between each latent factor and known RBPs, only LF2 has a significant overlap (27/298, *p* = 4.19 × 10^−6^, hypergeometric test). n.s.: not significant. **c** Heatmap showing normalized read counts of top 15 RBPs (rows) observed in LF2 for each sample (columns). Each gene is annotated according to its differential expression status between proximal-distal and dorsal-ventral-superficial-deep neurons (left vertical bar). Each sample is annotated according to region (top horizontal bar) and sample names are as in Fig. 2b. PD = proximal-distal neurons. **d** Heatmap showing the APA profiles inferred by QAPA, measured by proximal poly(A) site usage (PPAU), of the top-weighted 3′ UTRs (rows) for each sample (columns). **e** Bar-plot showing the proportion of top-weighted 3′ UTRs from each latent factor with genes known to localize to dendrites and axons^46^. Similar to **b**, when comparing the degree of overlap between each latent factor and known localization genes, only LF2 had a significant overlap (27/66, *p* = 3.85 × 10^−5^, hypergeometric test). Sample names are as in Fig. 2b. n.s.: not significant. **f** Scatterplot showing the latent factor 2 loadings (*y*-axis) for each 3′ UTR in ascending order from left to right (*x*-axis). Lengthening 3′ UTRs (indicated by negative loadings, Supplementary Fig. 3A) that belong to localized transcripts are represented by red dots. The bottom-right box shows genes with localized transcripts from the indicated region of the plot.

We next investigated LF2, which captures differences between neurons on the proximal-distal axis versus the other axes (i.e., dorsal-ventral and superficial-deep). Interestingly, APA is the primary layer contributing to this factor, followed by GE (Fig. 1b, see below). We first examined the contributions of GE by inspecting the top genes weighted by MOFA. GO analysis identified significantly enriched terms related to the ribosome and metabolism (Fig. 3a, FDR < 0.05, FDR-corrected hypergeometric test). To more generally assess whether the top genes regulated by APA, as weighted by MOFA, relate to functions associated with post-transcriptional regulation and translation, we asked whether these overlap with annotated RNA-binding proteins (RBPs) using data from Castello et al.^41^ and Ray et al.^42^. Interestingly, among the genes upregulated in the proximal-distal group, we observe significant enrichment for RBPs (27/298, *p* = 4.19 × 10^−6^, hypergeometric test; Fig. 3b). This enrichment is unique to LF2, as no such enrichment was detected among the top-weighted genes belonging to the other factors. Interestingly, the majority (*n* = 15) of the RBP genes encode ribosomal proteins, confirming the initial GO analysis results (Fig. 3c). Additionally, eight of these ribosomal protein genes show statistically significant upregulation in proximal and distal neurons compared to dorsal, ventral, superficial, and deep neurons ($\left| {{\mathrm{log}}_2\,\phi } \right| \, > \, 1s$, FDR < 0.05, where $\phi$ is the fold change in gene expression between these two groups).

These observations prompted the question as to whether additional ribosomal protein genes share a similar expression pattern and may have been missed in the above analysis. To address this, we examined the expression profiles of all annotated ribosomal proteins by compiling genes associated with GO terms containing the term “ribosomal subunit” (Supplementary Fig. 2). Interestingly, an additional 29 ribosomal protein genes were found to display upregulation in the proximal-distal versus dorsal-ventral-superficial-deep axes comparisons. These findings suggest that the composition of ribosomal components in pyramidal neurons in the proximal-distal axis is distinct from that of the dorsal, ventral, superficial, and deep pyramidal neurons, consistent with increasing evidence for cell-type- and condition-dependent quantitative and qualitative differences in the translation machinery^43^.

### A is coupled to lengthening of 3′ UTRs in the proximal-distal axis

Next, we investigated the contribution of APA in LF2. To assess APA, we used QAPA to measure proximal poly(A) site usage (PPAU), i.e., by quantifying the usage of proximal 3′ UTR sequences relative to all 3′ UTR isoforms of a gene. Similar to the observations for GE differences described above, the factor loadings for APA were strongly correlated with ΔPPAU changes between proximal-distal pyramidal neurons versus pyramidal neurons from the dorsal-ventral and superficial-deep regions of the hippocampus (Pearson correlation *R* = 0.9, $p \;< \;2.2 \times 10^{ - 16}$; Supplementary Fig. 3a). By examining the ΔPPAU values of the top-weighted 3′ UTRs, we observed that the majority of the changes involve lengthening of 3′ UTR sequences in proximal-distal neurons, compared to other pyramidal neurons (Fig. 3d and Supplementary Fig. 3b).

APA can elicit coupled regulatory changes in neurons, for example, by affecting where transcripts are localized for translation, and/or by controlling the steady-state GE levels of transcripts and corresponding protein products. To address whether the APA differences detected above are coupled with changes in steady-state GE, we performed a differential GE analysis on the corresponding transcripts using DESeq2. Consistent with previous studies indicating that APA is not significantly correlated with steady-state GE changes during neuronal differentiation^34,44,45^, we do not observe a correlation between ΔPPAU and changes in transcript levels between proximal-distal pyramidal neurons versus the other profiled neurons (Supplementary Fig. 3c). This observation suggests that APA represents a largely distinct regulatory layer with a more prominent role in pyramidal neurons that form the proximal-distal axis of the hippocampus.

To assess whether 3′ UTRs with APA changes could potentially be involved in coupled changes in mRNA localization in neurons, we determined the overlap between the genes from the top-weighted APA events of each latent factor with a previously reported set of transcripts from 2,550 genes found to be preferentially localized to the dendrites and/or axons of mouse hippocampal CA1 neurons^46^. Interestingly, only genes within LF2 significantly overlapped with this set of genes (27/66, *p* = 3.85 × 10^−5^, hypergeometric test; Fig. 3e, f). These results indicate that changes in the length of 3′ UTRs between the proximal-distal axis, relative to other hippocampal axes, are likely coupled to transcript regulation through differential localization^47^.

### Analysis of multiple layers of gene regulation facilitates identification of distinct drivers of neuronal specialization

**Fig. 4:**
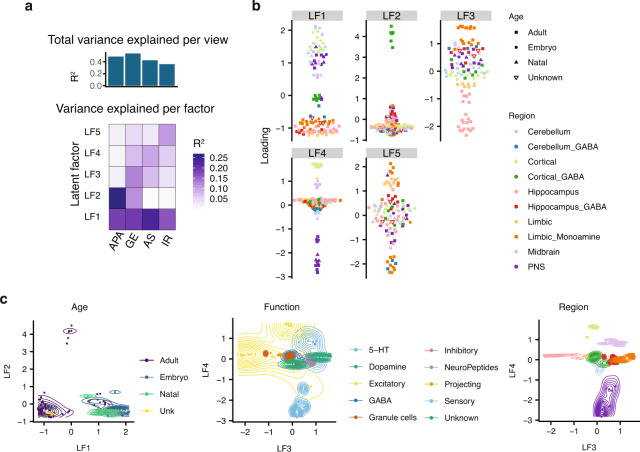
Expanded analysis of whole-brain reveals coordinated regulation by all layers. **a** Summary of latent factors inferred by MOFA and the contributions of each layer to each factor (see Fig. 1b for details). **b** Beeswarm plots illustrating the variation described by the latent factors inferred by MOFA (see Fig. 1c for description of Beeswarm plots). **c** Combined scatter and contour plots comparing the loadings for pairs of latent factors: LF1 versus LF2 (left), LF3 versus LF5 (middle and right). The contours indicate the density of cell types for a specific attribute. In particular, age is a highly correlated with LF1 (left), excitatory neurons are correlated with LF3 (middle), and PNS neurons are correlated with LF4 (right). unk = unknown.

We next expanded our analysis of GE, APA, and AS to assess the relative contributions of each of these regulatory layers to a more diverse set of neuronal populations distributed across the mouse nervous system, represented by more than one-hundred RNA-seq datasets. These neuronal samples were annotated on the basis of a range of phenotypic features including excitatory type, morphology, age, and brain region (Supplementary Data 2). Applying MOFA resulted in the inference of five latent factors, each of which have shared and unique contributions representing each regulatory layer (Fig. 4a). Importantly, surrogate variable analysis reveals that these results are independent of batch effects (Supplementary Fig. 4a, b, Supplementary Results, and Supplementary Methods) minimizing the potential impact of ratio-based metrics^48^. Similar to the hippocampal sub-region analysis, GE accounted for the most variance (*R*^2^ = 0.55). The other layers also contributed substantially to the observed variation (APA, *R*^2^ = 0.49; exonic AS, *R*^2^ = 0.44; and IR, *R*^2^ = 0.37). Furthermore, these factors describe variability consistent with the results from PCA (Supplementary Fig. 5a).

To further investigate the importance of interconnections between the analyzed regulatory layers, we overlapped each latent factor used with manually annotated phenotypic data associated with individual neuronal datasets (Supplementary Data 2). This analysis revealed that developmental stage was the major contributor to distinctions between neuronal populations (LF1 in Fig. 4b, c). However, whereas previous work focused on the role of GE in defining differences between neuronal subtypes^49^, our analysis suggests that each surveyed regulatory layer contributes substantially to neuronal specification, thus highlighting the contribution of multiple levels of regulatory coordination in neuronal development and specialization (Fig. 4a and Supplementary Fig. 5b). For example, APA differences predominantly characterize the separation of inhibitory cortical GABA interneurons from other neuronal subtype populations (LF2), GE differences drive the clustering of the excitatory neurons of the hippocampus and limbic system from other neuronal populations (LF3), and AS is the main regulatory layer separating populations of neurons from the central and peripheral nervous systems (LF4) (Fig. 4c). Altogether, this analysis reveals that through a combined analysis of multiple layers of gene regulation we can identify, in an unsupervised manner, distinct transcriptomic signatures that distinguish neuronal populations on the basis of age, brain region and electrophysiology.

### Post-transcriptional regulatory layers distinguish neuronal populations

**Fig. 5:**
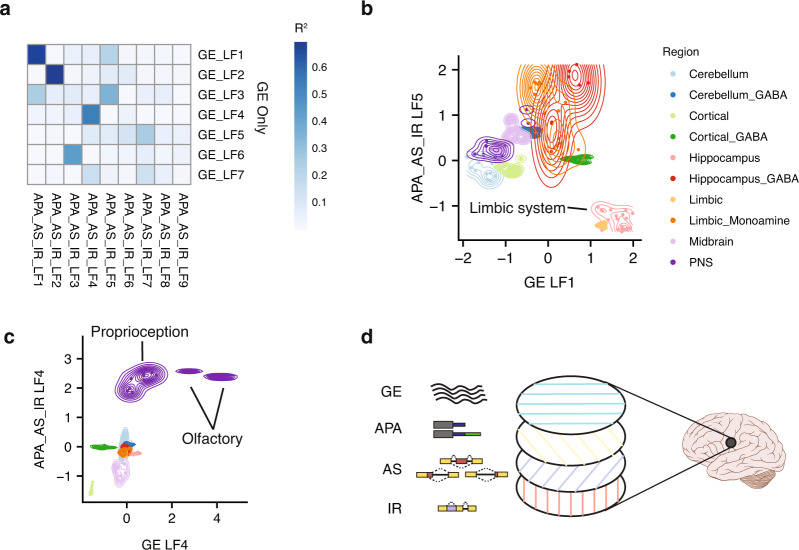
Variable contributions of transcriptomic regulatory layers to the definition of neuronal identity. **a** Heatmap showing the Pearson correlation (*R*^2^) between pairs of latent factor weights assigned to each sample inferred when only GE data is analyzed (*y*-axis) and when only APA_AS_IR data are analyzed (*x*-axis). Latent factors that are highly correlated indicate neuronal population groupings identified by GE data only, and independently by APA_AS_IR data. **b** Combined scatter and contour plot comparing the latent factor weights between GE-LF1 and APA_AS_IR-LF5. Both factors identify a cluster of hippocampal neurons. However, each factor distinguishes the neurons differently (see Fig. 4c for description of contour plots). **c** Comparison between GE-LF4 and APA_AS_IR-LF4. Both factors identify a cluster of peripheral nervous system (PNS) neurons. However, GE-LF4 specifically identifies a subset of PNS neurons belonging to the olfactory system. **d** Multiple “layers” of coordinated transcriptomic regulatory networks collectively and differentially contribute to establishing neuronal identity throughout the nervous system. Brain schematic by Patrick Lynch^78^, distributed under a Cresative Commons Attribution License.

Given the contributions of AS, APA, and IR to the latent factors defined in the whole-brain analysis, we next asked whether these post-transcriptional regulatory layers, independently of GE, can distinguish different properties of neurons. We therefore applied MOFA under two scenarios: one using data from only AS, APA and IR, and one using data from GE alone (Supplementary Fig. 5c; see Methods). To determine whether similar groups of neuronal subtype populations were identified in both situations, the latent factor weights assigned by MOFA for each neuronal subtype population cluster were used to compute pairwise correlations (Fig. 5a; see “Methods”). In line with a major role for post-transcriptional regulation in determining neuronal fate^11,50^, the majority of the neuronal populations identified by GE were also readily distinguished by MOFA using data derived from post-transcriptional regulatory layers alone (i.e., AS, APA, and IR).

Importantly, however, we observed that a number of neuronal subtype populations defined by AS, APA, and IR were not identified by the analysis of GE alone. In particular, AS, APA, and IR distinguished excitatory and inhibitory neurons of the limbic system, whereas GE data did not discriminate these classes of neurons (Fig. 5b). In contrast, when only GE data were used, olfactory system and the proprioceptor neurons clustered separately, whereas AS, APA, and IR were only able to distinguish neurons from the peripheral and central nervous system (Fig. 5c). This suggests that despite the importance of multi-layered regulatory networks in defining the properties of the nervous system, certain distinguishing characteristics of neuronal populations are more strongly associated with some layers than others. Thus, consistent with findings from analyzing neurons from different hippocampal regions described above, neuronal specificity more generally depends on the collective contribution of multiple regulatory layers, but each layer may have a more prominent relative contribution than other layers depending on the specific spatial and functional properties of a neuronal subtype population (Fig. 5d).

## Discussion

An important goal of transcriptomics research is to understand how different layers of gene regulation are integrated to contribute to functional transcript and protein diversity^51,52^. The mammalian nervous system is an important example illustrating this concept^10,11^. Previously, Cembrowski et al.^28^ used RNA-seq data to demonstrate a continuous gradient of GE changes across the dorsal-ventral axis of CA1 field of the hippocampus. Other studies have suggested that both continuous and discrete GE changes are involved in the specification of neuronal subtypes from different hippocampal regions^53,54^. In the present study, we analyzed RNA-seq data from mouse hippocampal neurons from Cembrowski et al.^28^ to generate profiles of GE, AS, and APA. The integration of these layers using MOFA^35^ revealed important sources of additional regulatory variation between different hippocampal neuronal subtypes.

A major focus of the present study was analyzing differences between pyramidal neurons in the CA1 proximal-distal axis versus neurons from other axes of the hippocampus. CA1 cells in the hippocampus display location-specific firing^55^ defined by differences in the bursting patterns of proximal versus distal CA1 neurons^56^, which may be required for animals to track their location in unfamiliar locations^55^. Proximal neurons are more sensitive to processing spatial memories (e.g., location of an object), whereas distal neurons are sensitive to non-spatial, temporal memories (e.g., features of an object)^57^. Our findings suggests that biological differences between proximal-distal axis neurons may primarily be driven by post-transcriptional regulatory factors^28^.

Unexpectedly, we observed that the underlying variation between proximal and distal neurons involves coordinated programs of GE and APA changes, involving the differential expression of ribosomal protein genes and differential 3′ UTR isoform usage of other gene sets, respectively. In the latter case, genes associated with 3′ UTR isoform changes significantly overlap genes encoding transcripts previously reported to localize to dendrites and axons^46^. This raises the question of how these two correlated patterns combine to specify spatially distinct neuronal functions. Previous studies have demonstrated extensive heterogeneity in ribosome composition and function (reviewed in ref. ^43^). These ‘specialized ribosomes’ enable spatial and temporal translational control of selective mRNAs under different physiological conditions and tissue types^43^. Moreover, this specialization is important in neurons, where dendrites and axons are distant from the cell body^58,59^. Although ribosome assembly generally occurs in the nucleolus, it has been observed that components of the translational machinery can be transported in the form of large ribonucleoprotein particles or granules, and select ribosomal protein mRNAs are locally synthesized^43^.

The localized translation of individual synaptic mRNA transcripts is highly regulated^41,51^. To efficiently carry out local protein synthesis, mRNAs are transported to subcellular neuronal compartments, where they are stored and translated on demand^26^. In many cases, localization is dependent on sequence elements encoded in 3′ UTRs. As such, differential selection of alternative 3′ UTR isoforms due to APA can regulate mRNA localization to neuronal compartments^26,60,61^. In summary, the results from our analysis of hippocampal RNA-seq data suggest coordinated functions of specialized local translation and localization of mRNAs to synaptic compartments occurring along the proximal-distal axis, principally through the combined contributions of GE and APA regulatory layers. Further research will be required to determine the specific functional roles of differential mRNA localization and translational control in defining proximal-distal hippocampal neurons.

Alternative splicing also plays an important role in the definition of specific neuronal subtypes^23,62^. For example, previous work has demonstrated neuronal-type-specific AS regulation in the cortex^22,63^. Expanding our analysis to multiple neuronal subtype populations across the nervous system in the present study revealed that AS is strongly correlated with various phenotypic features including the developmental stage, morphology, and type of excitatory neuron. Similar to the analysis of hippocampal neurons, these results further demonstrate the importance of integrating data from different post-transcriptional regulatory layers to capture variation that is not observed by GE differences alone.

Recent advances in the development of single-cell RNA-seq methods have afforded the identification of rare and novel cell types, and have contributed to the characterization of the extensive cell diversity of the brain^64,65^. However, to-date, these studies have primarily relied on measuring GE changes. As differential GE patterns between cell and tissue types are generally more conserved than differences at the level of post-transcriptional regulation^19,20^, single-cell RNA-seq studies relying on GE measurements alone likely underestimate cell-type diversity. While efforts to develop methods for detecting AS and APA from single-cell RNA-seq data have been described^66,67^, challenges remain, including technical limitations involving limited sequencing depth and read length^68^. It is also important to note that our results are based on analyzing cell types initially identified by gene expression markers and, therefore, probably also underestimate neuronal diversity driven by post-transcriptional regulation. Therefore, based in part on the findings of the present study, in the future it can be anticipated that additional spatially and functionally distinct neuronal subtypes will be defined by integrating measurements from multiple transcriptomic regulatory lawyers. Our results thus highlight the importance of developing cost-effective approaches to measure and incorporate the analyses of multiple regulatory layers when identifying and characterizing distinct neuronal subtypes using single-cell RNA-seq.

## Methods

### Datasets

The RNA-seq datasets used in this study were downloaded from the NCBI Gene Expression Omnibus (https://www.ncbi.nlm.nih.gov/geo/). For the mouse hippocampus analysis, the accession number is GSE67403^28^ (https://www.ncbi.nlm.nih.gov/geo/query/acc.cgi?acc=GSE67403, Supplementary Data 1). For the whole-brain analysis, a summary of the RNA-seq datasets and their accession numbers can be found in Supplementary Data 2.

A list of RBPs was obtained from the Supplemental Information of Castello et al.^41^ and from the CISBP-RNA database (http://cisbp-rna.ccbr.utoronto.ca/)^42^.

A list of mRNAs localized to dendrites and axons was obtained from the Supplemental Information of Cajigas et al.^46^.

A list of 1816 human transcription factors was obtained from Lambert et al.^36^. Mouse orthologs for each transcription factor were determined using Ensembl BioMart database (https://www.ensembl.org/biomart/martview).

### RNA-seq pre-processing

Initial quality control was performed using FastQC (http://www.bioinformatics.babraham.ac.uk/projects/fastqc/). To ensure accurate AS and IR quantification, when necessary datasets were combined to ensure a minimum depth of 40M paired-end reads. One amygdala sample (SRR2229946) was discarded from this analysis, as it appeared to be a distinct outlier in preliminary analysis by PCA.

### Gene expression analysis

To measure steady-state gene expression levels, Salmon^31^ was used to measure transcript abundance, based on mouse (mm10) GENCODE whole transcript annotations^69^. Gene-level abundance was then computed by adding the estimated read counts mapping to each transcript isoform using the R package tximport^70^. To perform differential gene expression analysis between pairs of neuronal subtypes, DESeq2^37^ was applied on the gene-level counts estimated from above. Differentially expressed genes are defined as those with a $|{\mathrm{log}}_2\,\phi | \, > \, 1$ and FDR < 0.05, where $\phi$ is the fold change between a pair of subtypes.

For downstream analysis, variance-stabilized read counts were computed using the DESeq2 function varianceStabilizingTransformation()^37^. After removing genes with a median read count of <5, the top 5000 most variably expressed genes based on standard deviation were retained for downstream analysis.

### Alternative splicing analysis

To comprehensively detect and quantify AS and IR events, we used the VAST-TOOLS multi-module analysis pipeline (https://github.com/vastgroup/vast-tools), as previously described^17^, as well as Whippet (https://github.com/timbitz/Whippet.jl), a lightweight algorithm for event detection and quantification^33^.

VAST-TOOLS was used to detect and quantify AS and IR events in the hippocampal datasets, as described previously^71^. Briefly, reads were initially mapped to genome assemblies (mm9) using Bowtie, (–m 1 –c 2 parameters) with reads that mapped to the genome discarded for AS/IR quantificatio. For AS, unique EEJ (exon–exon junction) libraries were generated to derive measurements of exon inclusion levels using the metric “Percent Spliced In” (PSI). This utilized all hypothetically possible EEJ combinations from annotated and de novo splice sites, including both cassette, mutually exclusive and microexon events. For intron retention (IR), a comprehensive set of reference sequences comprising each IR event was used: two exon-intron junctions (EIJs), intron mid-point sequences, and EEJs formed by intron removal^72^. Each IR event requires multiple reads mapping to both the EIJ and the intron mid-point sequence, as described previously^72^.

Whippet was used with default settings to analyze RNA-seq data from the whole-brain datasets (see Supplementary Data 2). To create splice graphs required for Whippet quantification, mm10 genome annotation files were extracted from the Ensembl database. Whippet was used for AS to quantify all combinations of EEJs, including cassette, mutually exclusive, microexon events, and acceptor and donor splice sites, as well as for IR to quantify all combinations of EIJs.

### Differential splicing analysis

Differential identification of percentage splicing in (PSI) for AS events or percentage intron retained (PIR) for IR events were calculated using the VAST-TOOLS diff module (–minReads = 10), as described previously^71^. Events were screened for sufficient read coverage by keeping those with “OK/SOK” quality designation in 60% of samples.

### Alternative polyadenylation analysis

APA analysis was performed using QAPA (https://github.com/morrislab/qapa) as previously described^34^, except that Salmon was used to quantify 3′ UTR transcript abundance. Briefly, a mouse (mm10) 3′ UTR reference library was constructed using GENCODE gene model annotations. To obtain a more comprehensive set of 3′ UTRs, the library was augmented by additional poly(A) site annotations, which added new 3′ UTR isoforms not characterized in GENCODE, or else updated the 3′ ends of existing isoforms. To avoid the possibility of converging genes that have overlapping non-strand-specific RNA-seq reads, converging genes with distal 3′ UTR poly(A) sites within 500 nt of each other were excluded. Genes with 3′ UTR lengths of <100 nt were also excluded.

To further filter events for MOFA analysis (see below), the following steps were performed. First, genes with total expression of at least 3 transcripts per million (TPM) in 22 or more (out of 24) samples were retained. This ensures that genes are expressed in the majority of samples studied. Second, these genes were further filtered for those whose proximal 3′ UTR is expressed by at least 1 TPM in six or more samples. This ensures that there are examples of APA where the proximal 3′ UTR is expressed (in comparison to other samples). Finally, we filtered for 3′ UTRs with a |∆PPAU| > 20 between one or more pairs of cell types, where ∆PPAU is defined as the difference between the median PPAU of two cell types.

### Principal component analysis

Principal component analysis (PCA) was performed on mean-centered values using the R function prcomp().

### Inference of hidden factors from combined data sources

MOFA^35^ was used to infer the shared sources of variation between multiple data types. To prepare for model training, four sets of regulatory layers were used: GE, APA, AS, and IR. The AS layer included all exon-based events, including cassette exons and alternative 5′ and 3′ splice sites, while IR was treated as a separate layer. Each layer was filtered to include features with sufficient variation across samples, as summarized in Table 1 and described above. Training of the model was carried out using the following options: for hippocampal analysis, DropFactorThreshold = 0, tolerance = 0.01, maxiter = 6000; for whole-brain analysis, DropFactorThreshold = 0.02, tolerance = 0.01, maxiter = 5000.

**Table 1:** Filtering criteria for each regulatory layer.

Layer	Number of training examples	Selection criteria
GE	5000	Top 5000 most variably expressed genes based on median of normalized read counts
AS	762	Splicing events with $|{\Delta}{\mathrm{PSI}}|\; > \;20$ between at least one pair of cell types
IR	1526	*(same as above)*
APA	1513	Multi-UTR genes $|{\Delta}{\mathrm{PPAU}}| \ge 20$ between at least one pair of cell types

Each layer was pre-processed to retain the genes or AS/IR/APA event with the most variation.

*PPAU* proximal poly(A) site usage, *PSI* percent spliced-in.

To quantify the contribution of each sample in a latent factor, MOFA assigns factor weights such that similar samples (in terms of the variance explained in the low-dimensional latent space) will have similar weighting. To identify similar latent factors between the GE-only and AS, APA, and IR-only models (Fig. 5a and Supplementary Fig. 4c), pairwise Pearson correlations were computed between latent factor weights using the R function cor.test().

To quantify the contribution of each feature in a latent factor, MOFA assigns factor loadings that indicate its degree of correlation with the described latent factor pattern. The loadings were then transformed into *Z*-scores and queried for features with the top positive and negative scores.

### Functional enrichment analysis

Functional enrichment analysis was performed using g:Profiler^73^. Genes enrichment sets were compared to a background of expressed genes. Structured controlled vocabularies from Gene Ontology, as well as information from the curated KEGG and Reactome databases were included in the analysis. Only functional categorizes with more than five members and fewer than 2000 members were included in the analysis. Significant terms were summarized using Enrichment Map^74^ in Cytoscape^75^.

### Protein features

Overlap of AS exons with extracellular regions and transmembrane domains were assessed using two approaches. (1) Annotation of protein in Uniprot for extracellular protein expression; (2) Analysis by TMHMM (Transmembrane hidden markov model, http://www.cbs.dtu.dk/services/TMHMM/) for extracellular location of amino acid residues within a transmembrane protein.

### Ribosomal protein paralogs

A list of ribosomal protein paralogs were downloaded from the Ensembl Biomart database, using Ensembl Genes 94 and mouse genes GRCm38.p6 databases. Genes were filtered for those associated with GO terms containing the keyword “ribosomal subunit”.

### Localization data

A list of 2550 genes reported to be localized in dendrites and axons was obtained from Cajigas et al.^46^. This was compared with the top APA features in each factor ($\left| Z \right| \le 1.96$, $p \le 0.05$). To test for statistically significant enrichment, a hypergeometric test was performed using the R function phyper().

### Reporting summary

Further information on research design is available in the Nature Research Reporting Summary linked to this article.

## Supplementary information

Supplementary Information

Supplementary Data 1

Supplementary Data 2

Reporting Summary

Description of Additional Supplementary Files

## Data Availability

All relevant data accession IDs used in study are referenced in Supplementary Data. Pre-processed datasets for gene expression, alternative splicing and intron retention, and alternative polyadenylation and other supporting data are available at 10.6084/m9.figshare.13141328^76^. All data is available from the corresponding author upon reasonable request.

## Code availability

The data analysis R scripts used in this analysis are available at 10.6084/m9.figshare.13141328^76^.
