# Supplementary material for: Differential contribution of transcriptomic regulatory layers in the definition of neuronal identity
Source: Nat Commun. 2021 Jan 12;12:335. doi: 10.1038/s41467-020-20483-8 (PMC7804943; doi:10.1038/s41467-020-20483-8)
Supplement: Supplementary file 1 — Supplementary Information [file 41467_2020_20483_MOESM1_ESM.pdf]

# Supplementary Results

As the samples from the whole brain analysis used in this manuscript were collected from several studies and processed by different groups, we wanted to verify that major batch effects did not confound our analysis. To investigate this, we performed surrogate variable analysis (sva) on gene expression count data to infer a set of surrogate variables from the data, which represents possible latent variation while maintaining associations with the variables of interest [1]. In this case, the variables of interest were region and age (see Supplementary Methods). Reassuringly, we did not find any surrogate variables that strongly correlated with the origin of study (Supplementary Fig. 4a). This suggests that none of the studies had a major influence due to batch effects. However, two of the surrogate variables (SV4 and SV5) were observed to capture variation found in the hippocampus. This may actually represent biological variation given the heterogeneity found within the hippocampal samples, as further discussed below.

It is also possible that technical variation is captured by the latent factors inferred by MOFA. In these cases such latent factors can be identified and removed from the analysis. To confirm that the major latent factors described in our study did not strongly correlate with the surrogate variables identified by sva, we performed pairwise correlations between each latent factor and surrogate variable. Indeed, we observed that none of the pairs of variables were strongly correlated (Supplementary Fig. 4b).

We did not incorporate the surrogate variables into our analysis, as sva is often not suitable when aim of study is to characterize the heterogeneity of different biological subgroups [2]. This is because often some of the surrogate variables may inadvertently capture biological variation. The removal of such variables could potentially lead to inconsistent results. As a result of this caveat, we did not incorporate the surrogate variables into our analysis, but instead used it to inspect the validity of the latent factors inferred by MOFA.

# Supplementary Methods

## Surrogate variable analysis

Surrogate variable analysis was performed on the GE layer using the svaseq() function from the R package sva[3]. Surrogate variables were inferred from normalized read counts of the GE layer. To

construct the model matrix for svaseq(), the Region and Age attributes (Supplementary Data Table 2) were included in the model as variables of interest [e.g. model.matrix(~Region + Age, ...)]. For the null model, only the intercept was used [e.g. model.matrix(~1, …)].

## References:


1. Leek, J. T. & Storey, J. D. Capturing Heterogeneity in Gene Expression Studies by Surrogate Variable Analysis. *PLoS Genet* **3**, (2007).
2. Leek, J. T., Johnson, W. E., Parker, H. S., Jaffe, A. E. & Storey, J. D. The sva package for removing batch effects and other unwanted variation in high-throughput experiments. *Bioinformatics* **28**, 882–883 (2012).
3. Leek, J. T. *et al. sva: Surrogate Variable Analysis*. (2018).
4. Reimand, J. *et al.* g:Profiler -- a web server for functional interpretation of gene lists (2016 update). *Nucleic Acids Research* **44**, W83–W89 (2016).
5. Conway, J. R., Lex, A. & Gehlenborg, N. UpSetR: an R package for the visualization of intersecting sets and their properties. *Bioinformatics* **33**, 2938–2940 (2017).
6. Cembrowski, M. S. *et al.* Spatial Gene-Expression Gradients Underlie Prominent Heterogeneity of CA1 Pyramidal Neurons. *Neuron* **89**, 351–368 (2016).

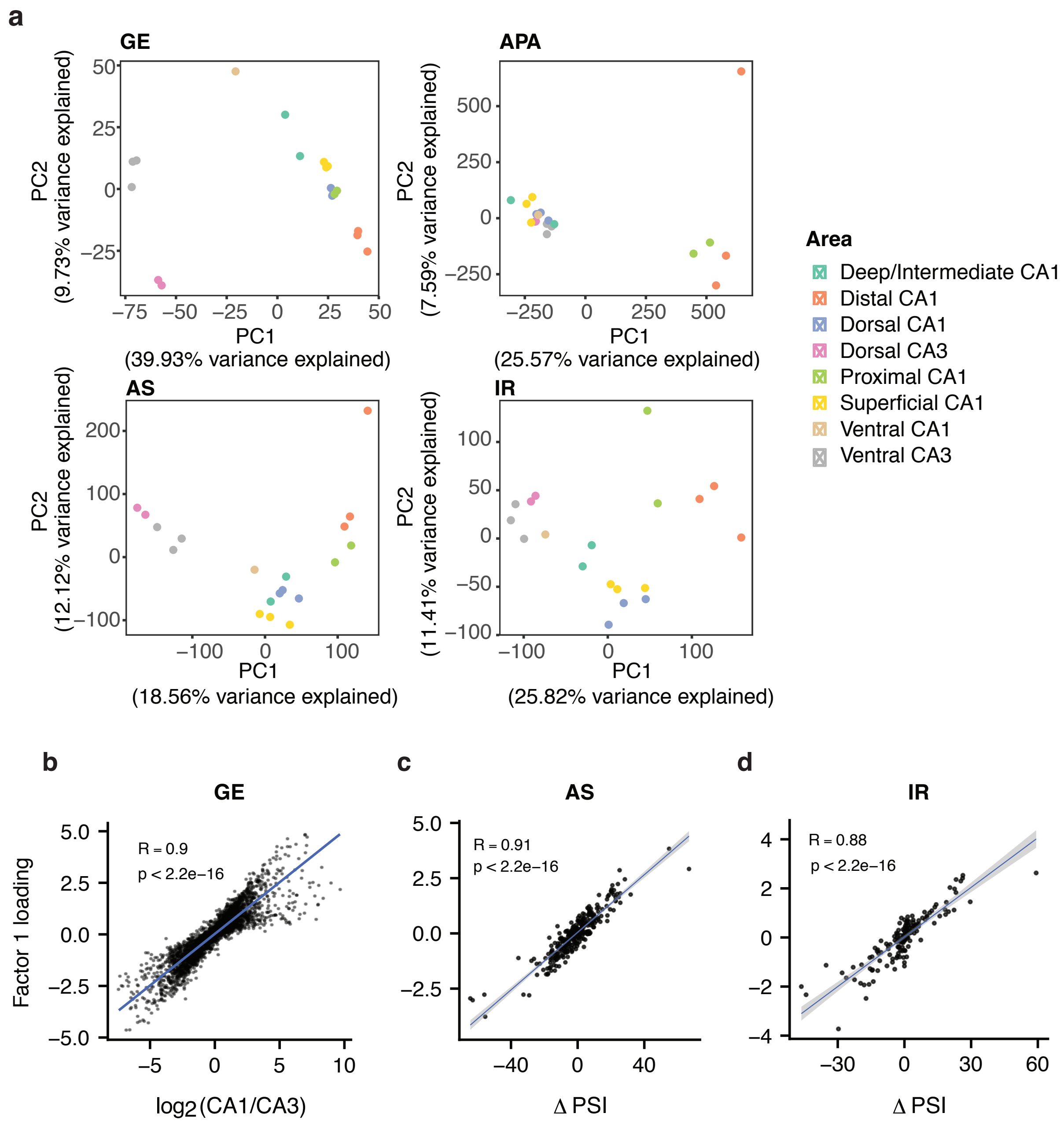


**Supplementary Figure 1 – Multi-layer analysis of hippocampal subtypes**

**(a)** Principal component analysis (PCA) of hippocampal neuronal cell type reveals variation explained by different regulatory layers. Each layer was analyzed independently and the first two principal components are visualized as scatterplots: Gene expression or GE (top-left), alternative polyadenylation or APA (top-right), alternative splicing or AS involving cassette exons and alternative 5' and 3' splice sites (bottom-left), and intron retention or IR (bottom-right). Each data point represents an RNA-seq sample. Points are labelled according to the sample's region within the hippocampus (colour).

**(b)** Comparing factor loadings with metrics for differential regulation. Scatterplots comparing LF1 loadings of features from the GE layer with metrics for differential regulation of each respective layer. (y-axis) were compared with the log2 fold change in gene expression between CA1 and CA3 subfields (x-axis). Pearson correlation (R = 0.90, p < 2.2 x 10-16) computed using the R function cor.test().

**(c)** Loadings from the AS layer were compared with the change in Percent Spliced-In (PSI) values between CA1 and CA3. Pearson correlation (R = 0.91, p < 2.2 x 10-16) computed as in (b).

**(d)** Similar to (c), but using loadings from the IR layer. Pearson correlation (R = 0.88, p < 2.2 x 10-16) computed as in (b).

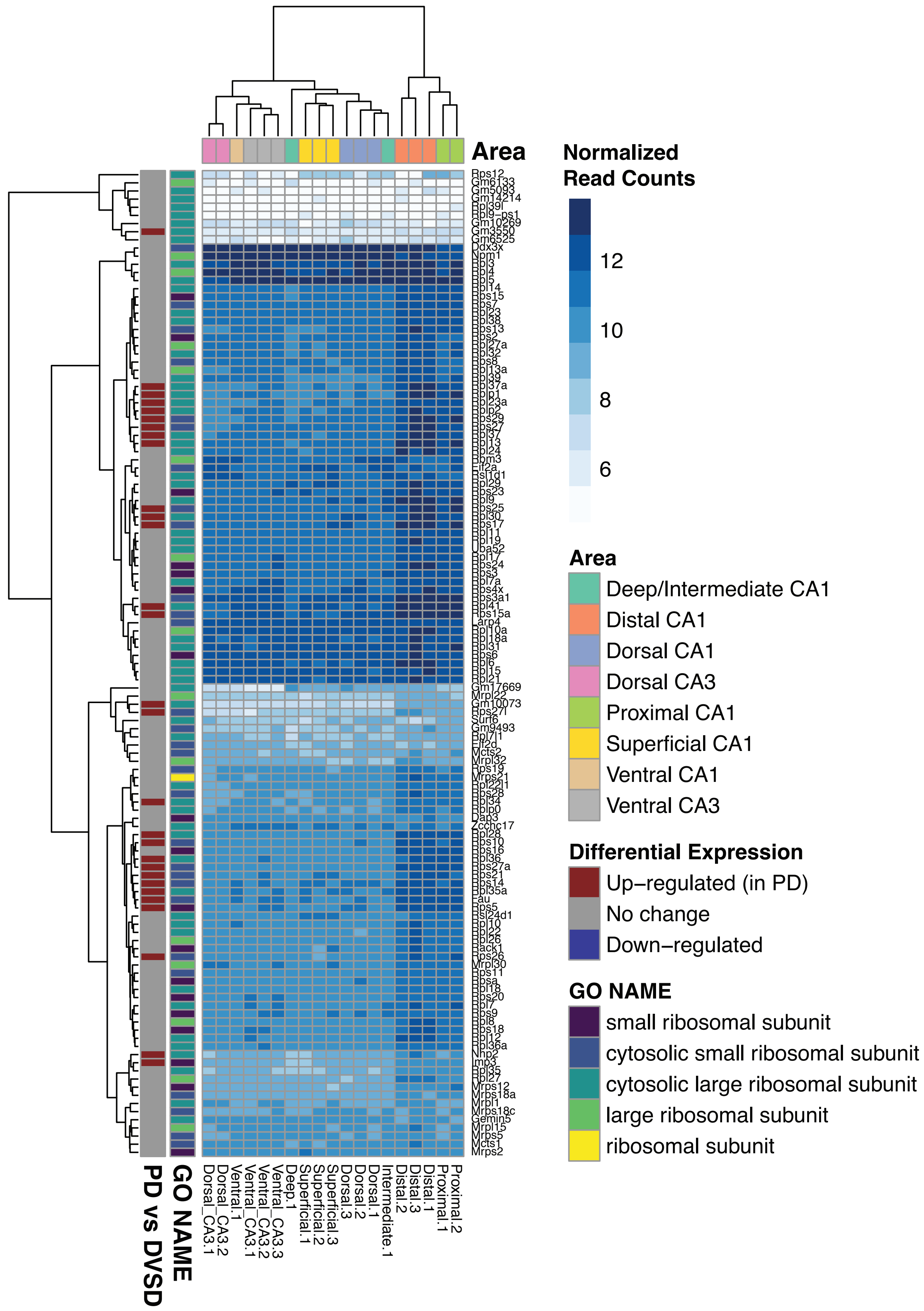


**Supplementary Figure 2 – Gene expression profiles of 144 ribosomal proteins.**

Heatmap showing normalized read counts of ribosomal proteins. Samples indicated on the x-axis are annotated according to area (top horizontal colour bar). Ribosomal proteins indicated on the y-axis are annotated according to their differential gene expression status between proximal-distal (PD) group and dorsal-ventral-superficial-deep (DVSD) group, and associated GO term. For the former, genes with statistically significant differential up- or down-regulation are indicated by red and blue, respectively (DESeq2, |log_2 ϕ|>1, FDR < 0.05, where φ is the fold change). Rows and columns are clustered using hierarchical clustering using complete-linkage method and Euclidean distance.

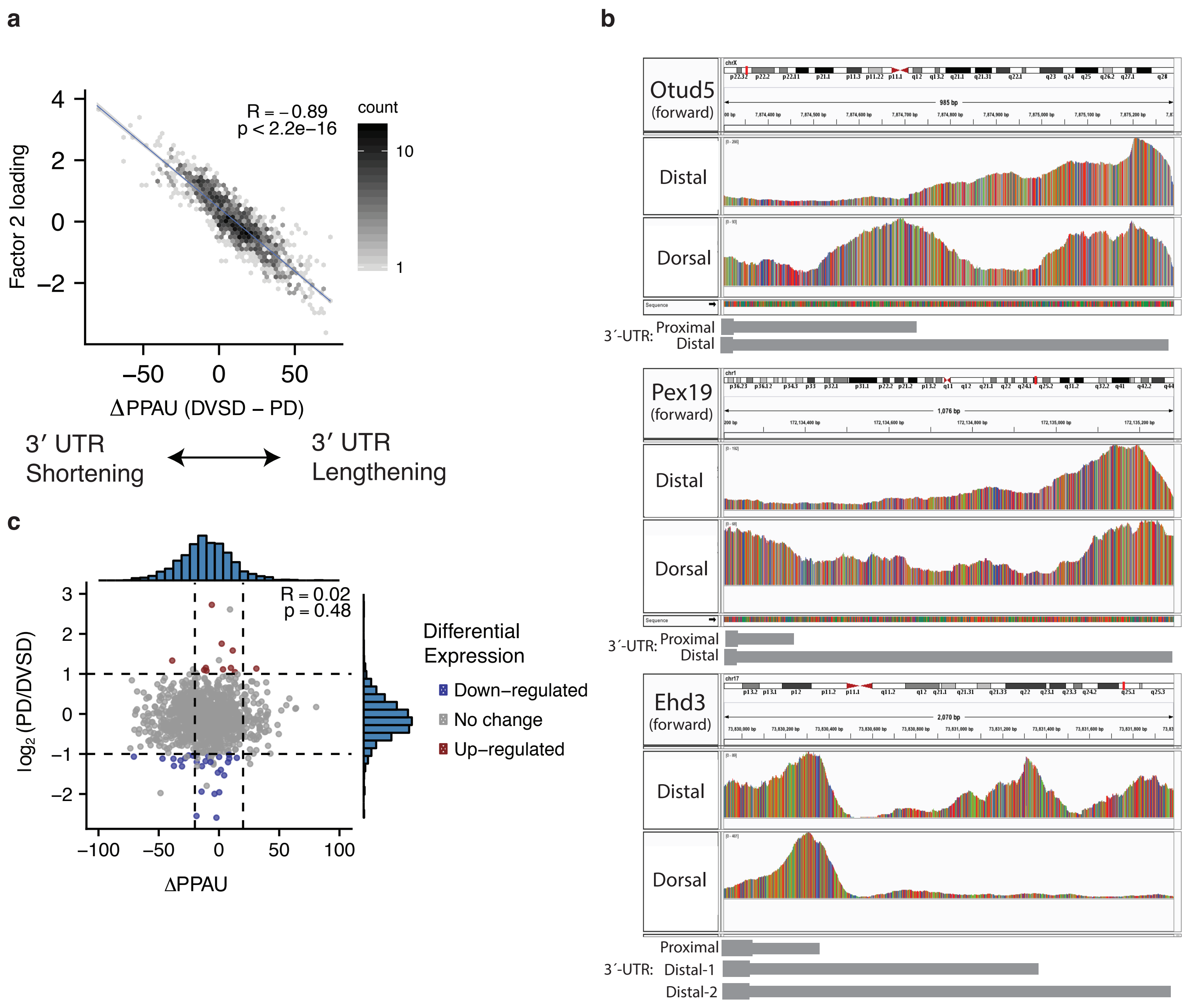


**Supplementary Figure 3 – Latent Factor (LF) 2 explains differences in proximal-distal axis versus neurons in other axes**

**(a)** Scatterplot comparing LF2 loadings (y-axis) of alternative polyadenylation (APA) features (e.g. 3' UTRs) with the change in proximal poly(A) site usage (ΔPPAU) values between the proximal-distal (PD) and dorsal-ventral-superficial-deep (DVSD) groups (x-axis). Positive ΔPPAU values correspond to 3' UTR lengthening, while negative values correspond to 3' UTR shortening. Pearson correlation (R = -0.89, p < 2.2 x 10-16) computed using the R function cor.test().

**(b)** Integrative Genome Viewer (IGV) browser (mm10) views displaying the lengthening of 3´-UTR sequences in distal neurons (top) compared dorsal neurons (bottom) of the mouse hippocampus. Bam coverage of aligned reads is displayed for three selected genes from the top ranked 100 APA events identified by MOFA analysis. The distal and proximal 3´-UTR structures are illustrated below the browser display.

**(c)** Scatterplot comparing ΔPPAU (x-axis) with their corresponding gene expression change between PD and DVSD groups. Genes with statistically significant differential up- or down-regulation are indicated by red and blue dots, respectively (|log_2 ϕ|>1, FDR < 0.05, where ϕ is the fold change). Dotted horizontal lines indicate log_2 ϕ thresholds (-1, 1), while dotted vertical lines indicated ΔPPAU thresholds (-20, 20). Pearson correlation computed using the R function cor.test().

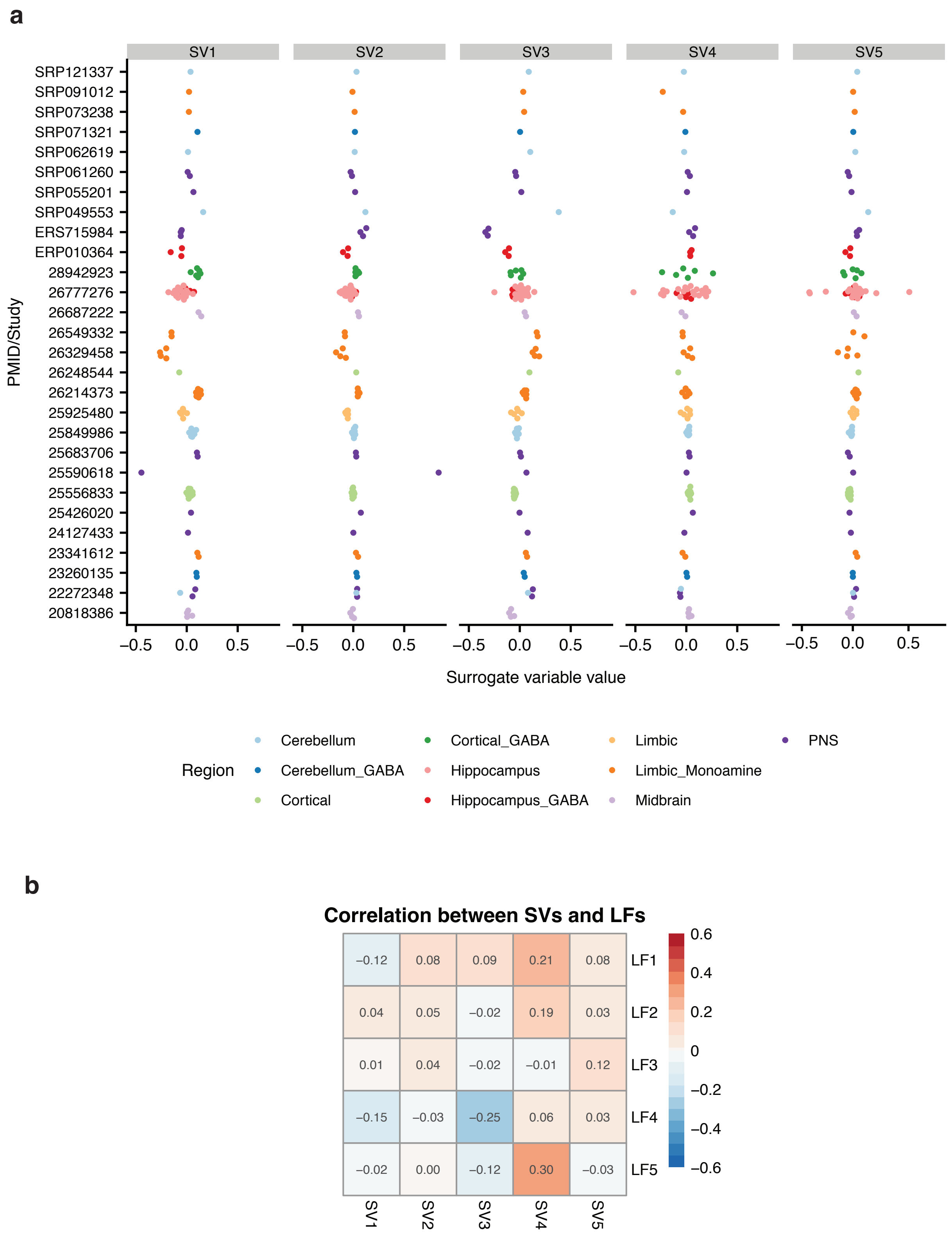


**Supplementary Figure 4 – Surrogate variable analysis of expression counts do not reveal significant batch effects**

**(a)** Beeswarm plots (see Fig. 1c for definition) illustrating the scores of five surrogate variables (SV1 to SV5; x-axis) grouped by the origin of study (y-axis). Each study is labelled according to its Pubmed ID (PMID) or accession number (Supplementary Data Table 2). Samples are coloured according to their annotated region. See Fig. 1c for description of Beeswarm plots.

**(b)** Heatmap summarizing the correlation between the surrogate variable scores and MOFA-inferred latent factors scores. Pearson correlation computed using the R function cor.test().

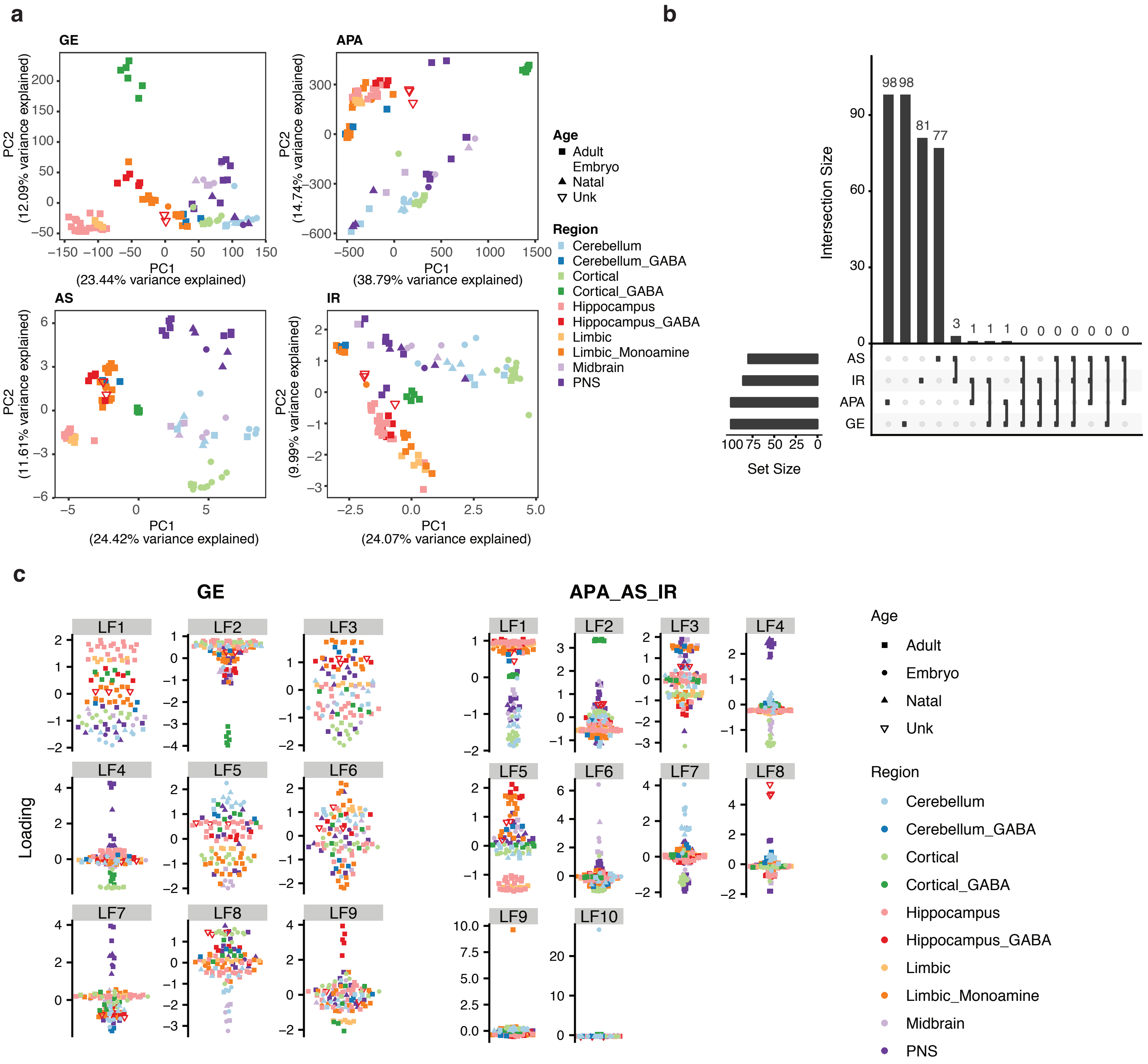


**Supplementary Figure 5 – Multi-layer analysis of neuronal subtypes across the mouse brain**

**(a)** Whole brain neuronal cell type variation explained by each regulatory layer. Principal component (PC) analysis of an expanded cohort of neuronal cell types across the nervous system. Similar to Fig. S1, each layer was analyzed independently. Each data point represents an RNA-seq sample. Points are labelled according to the sample's region within the hippocampus (colour) and age (shape). GE = gene expression; APA = alternative polyadenylation; AS = alternative splicing; IR = intron retention; Unk = unknown. PNS = peripheral nervous system

**(b)** An UpSet plot[5], which describe the overlap of sets equivalent to Venn diagrams. Each vertical bar along the y-axis indicates the number of overlapping genes between the layers indicated by the black dot(s) below. The UpSet plot shows that the overlap between the top weighted features from each layer in latent factor (LF) 1 represents a distinct (and non-overlapping) layer of regulation. The top 100 features by absolute LF1 weight for each layer were compared based on the gene source. For example, the genes that 3' UTRs belonged to in the APA layer were compared to genes from the GE layer.

**(c)** Comparison of MOFA models trained with and without GE. Beeswarm plots illustrating the factor loadings for MOFA models trained on GE data only (left) and with only data from post-transcriptional regulatory layers (right). See Fig. 1c for description of Beeswarm plots.
