# Supplementary material for: Differential contribution of transcriptomic regulatory layers in the definition of neuronal identity
Source: Nat Commun. 2021 Jan 12;12:335. doi: 10.1038/s41467-020-20483-8 (PMC7804943; doi:10.1038/s41467-020-20483-8)
Supplement: Supplementary file 5 — Description of Additional Supplementary Files [file 41467_2020_20483_MOESM5_ESM.pdf]

Description of Additional Supplementary Files

File Name: Supplementary Data 1
Description: Summary of RNA-seq samples for the mouse hippocampus dataset (Cembrowski et al., 2016)6

File Name: Supplementary Data 2
Description: Summary of RNA-seq samples for expanded analysis of mouse nervous system."
